# Assessment of an Anaerobic Membrane Bioreactor (AnMBR) Treating Medium-Strength Synthetic Wastewater under Cyclical Membrane Operation

**DOI:** 10.3390/membranes11060415

**Published:** 2021-05-31

**Authors:** Ahmet E. Uman, Robert A. Bair, Daniel H. Yeh

**Affiliations:** 1 Department of Civil and Environmental Engineering, University of South Florida, Tampa, FL 33617, USA; rbair@usf.edu (R.A.B.); dhyeh@usf.edu (D.H.Y.); 2 Bioengineering Department, Istanbul Medeniyet University, Istanbul 34000, Turkey

**Keywords:** AnMBR, medium-strength synthetic wastewater, ultrafiltration, NSSS

## Abstract

A lab-scale (6.2 L) anaerobic membrane bioreactor combined with a tubular, cross-flow, PVDF ultrafiltration membrane was developed and operated to assess the long-term fouling behavior of a cyclically operated anaerobic membrane bioreactor (AnMBR). The AnMBR was operated at 35 ± 1 °C for 200 days with a synthetic influent of 501 mg·L^−1^ COD to mimic municipal wastewater. The system exhibited high treatment performance with an average COD removal efficiency of 86.5 ± 6.4% (*n* = 20) and an average permeate COD concentration of 63.9 ± 31.1 mg·L^−1^. A clear permeate with an average turbidity of 0.6 ± 0.2 NTU, was achieved. Permeate TN and TP concentrations were 22.7 ± 5.1 mg·L^−1^ and 6.9 ± 2.0 mg·L^−1^ corresponding to removal efficiencies of 20.6% and 49.3%, respectively, likely due to membrane rejection of particulate, colloidal, and organic fractions. A stable membrane flux of 4.3 L.m^−2^.h^−1^ (LMH) was maintained for 183 days without gas-lift, gas sparge, or chemical cleaning. Cyclical operation with frequent relaxation (60 s for every 30 min of the permeate production run) and periodic permeate backwash (15 s for every 186 min) maintained stable membrane operation with an average TMP of 0.25 bar and a fouling rate of 0.007 kPa/h for the entire operating period. The comparison revealed frequent backwashing and relaxation is a sustainable strategy for operation of the AnMBR.

## 1. Introduction

There is growing interest in the sanitation industry on using more sophisticated decentralized wastewater (WW) treatment technologies to achieve objectives including onsite water recycling, off-grid operation, and nutrient recovery. Technologies capable of meeting these objectives while requiring minimal capital and operational costs can help meet the immediate sanitation needs of marginalized communities who currently lack access to adequate sanitation. These technologies, which are mostly still in development, are characterized in the recently developed ISO 30500 guidelines for non-sewered sanitation systems (NSSS) [1]. ISO 30500 establishes specific criteria for system reliability, safety, and effluent quality for these decentralized treatment technologies. Membrane bioreactors (MBRs) are an appealing technology that can meet many of the criteria set forth by ISO 30500. MBRs, which combine biological treatment with micro or ultra-filtration, enable process intensification, and generate a reliable high-quality effluent. MBRs have been used for decentralized WW reuse and sewer mining at the full-scale. However, these applications have mostly been in developed countries with reliable electricity and water infrastructure [2,3,4]. Indeed, most MBR research and applications have been with aerobic systems, which consume a great deal of electricity due to aeration. Aerobic systems also produce large quantities of waste biomass, which would be particularly challenging to manage at the household or community level. Biomass management would be especially challenging in areas lacking the financial resources for frequent desludging and other system maintenance.

Anaerobic MBRs (AnMBR) operate by a similar principle but leverage the advantages of anaerobic digestion. AnMBRs are able to treat WW without aeration, can produce energy in the form of biogas, and produce significantly less biosolids when compared to their aerobic counterparts [5,6,7]. A lab-scale study indicated that the operational cost of an AnMBR treating municipal WW could be a third of that of a similarly sized aerobic MBR (AeMBR) [8]. Similarly, a study that used the combined approach of life cycle analysis (LCA), life cycle costing (LCC), and steady-state modeling showed that an AnMBR was a more sustainable option compared to an AeMBR in terms of capital and operating expenditures (CAPEX/OPEX) when treating moderate to high-strength urban WW [9]. Another possible benefit is that AnMBRs convert particulate and organically-bound nutrients into soluble nitrogen (N) and phosphorous (P). The membrane-filtered effluent can then be reused through agricultural applications such as fertigation. Almost complete nutrient solubilization was observed in a 100-day study by Prieto et al. [10] where 96% of the total N and 93% of the total P in the influent were liberated to inorganic forms (NH_4_^+^ and PO_4_^2−^). Another study showed that it is possible to grow vegetables directly from AnMBR effluent in small, decentralized systems. This study observed that in terms of plant health indicators, AnMBR effluent was comparable to commercial fertilizers, showing the suitability of AnMBR effluent for fertigation in hydroponic systems [11].

AnMBRs have a high potential of meeting many of the ISO 30500 criteria; however, operational and environmental issues such as membrane fouling, dissolved methane recovery, and effluent nutrient management remain. Among these, membrane fouling remains a top priority as research shows current fouling mitigation strategies constitute a major energy demand for AnMBRs [12,13]. To address this issue, numerous invasive and noninvasive strategies have been suggested and examined. These methods include: quorum quenching; filamentous bacterial population control; membrane scouring using gas; the addition of activated carbon; membrane vibration and rotation strategies; various filtration cycles incorporating relaxation and/or backwashing; chemical cleaning using acids, bases, and oxidants; the addition of adsorbents and coagulants such as FeCl_3_, Al_2_(SO_4_)_3_, and PAC (polymeric aluminum chloride); and pretreatment processes integrated with AnMBRs or hybrid AnMBRs [12,14,15,16]. When taken separately or together, these strategies have been able to reduce the fouling propensity of their respective systems. However, most research on biofouling mitigation has been conducted on submerged AnMBRs as opposed to external membranes modules. It is impractical and inadvisable to directly compare the results from one type of configuration to another. 

Historically, many studies focused on submerged systems due to their lower energy requirements compared to external configurations. External membranes tend to have higher energy requirements as the reactor contents must be pumped to the membrane module and to provide a cross-flow velocity (CFV) high enough to scour the membrane surface. The energy difference between the two configurations can be substantial. An estimate of the power consumption of some submerged systems ranged between 0.69–3.41 kWh/m^3^, while external systems ranged from 3 to 7.3 kWh/m^3^ [17]. However, external membranes are valuable from an operations perspective as they are easier to service and allow for a less complex reactor design. The complex reactor design required by submerged AnMBRs precludes them from being used in small-scale systems that require minimal maintenance and lower cost reactors. In addition, the generalization that submerged systems have lower energy demands is not always true, as some external configurations have low energy consumption rates of 0.23 to 0.48 kWh/m^3^ [17]. These rates were achieved by operating at a low CFV. Many of the fouling control strategies that have been suggested require increasing system complexity, such as with gas-lift and scouring, or require regular consumables such as powdered activated carbon and granular activated carbon (PAC/GAC) additions. Approaches that increase system complexity or require additional consumables are inherently less desirable for decentralized systems. Methods that use existing hardware to minimize fouling in AnMBRs are preferred as they do not increase system complexity and do not require additional consumables. Incorporating low CFV along with low energy strategies such as membrane relaxation, backwashing, and careful cycling between them will likely lead to a long-term sustainable membrane performance with a low energy requirement.

The objectives of our study are as follows: (1) establish a stable long-term flux in an AnMBR with an external membrane utilizing cyclical operation (production cycle with frequent relaxation and periodic backwash) and low CFV for fouling control; (2) compare the fouling propensities of this study to those of a gas-lift design previously operated in our lab; (3) investigate overall AnMBR performance in medium-strength municipal WW treatment scenarios for chemical oxygen demand, total phosphorous, and total nitrogen (COD, TP, and TN) removals and permeate quality in order to examine the feasibility of meeting ISO 30500 standards.

## 2. Materials and Methods

### 2.1. Experimental Setup and Inoculum

**Figure 1:**
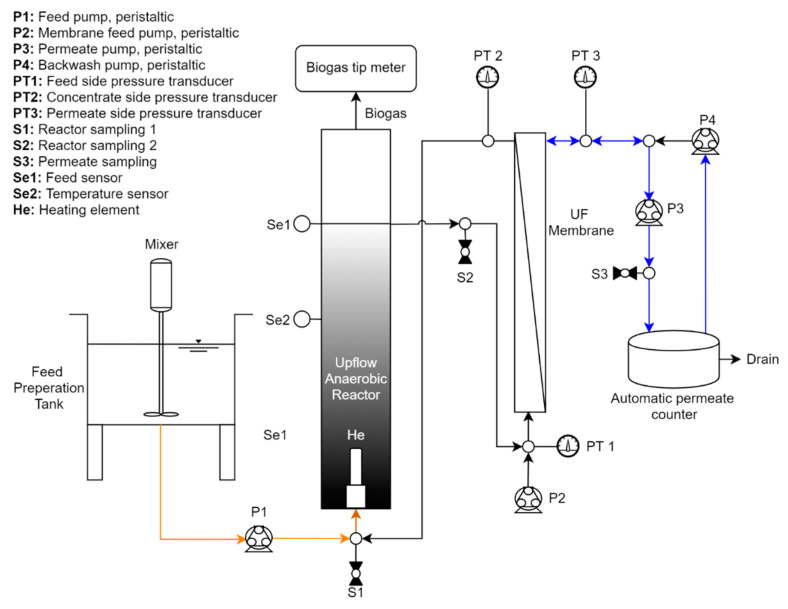
Schematic of the 6.2 L upflow anaerobic membrane bioreactor.

The laboratory-scale AnMBR consisted of an upflow anaerobic membrane bioreactor with a liquid volume of 6.2 L and a 1.2 L gas headspace (Figure 1). The custom-built ultrafiltration (UF) membrane module contained 4 tubular (1.1 m long, 5.2 mm ID, inside-out filtration) polyvinylidene fluoride (PVDF) membranes with a nominal pore size of 0.3 µm and a total membrane area of 0.075 m^2^ (Pentair X-Flow; Enschede, The Netherlands). For heating, a stainless-steel aquarium heater was inserted at the bottom of the reactor where the influent was fed. The temperature of the reactor was continuously monitored using inline sensors. The membrane module was equipped with three pressure transducers placed along the feed, concentrate, and permeate lines for monitoring of the transmembrane pressure (TMP). Sensor data were collected using HOBOware (Onset Computer Corporation; Cape Cod, MA, USA) software. For membrane circulation, permeate production, and backwashing, four peristaltic pumps were used (Cole-Parmer; Vernon Hills, IL, USA).

The following equations are pertinent to the cyclical membrane operation:

J_net_ = (Q_e_/A_m_) ÷ 24 h/d (1)

t_CF_ = 24/t_PT_ (2)

J_ins_ = J_net_ × t_CF_ (3)

t_PT_ = 6 × t_P_ × n_C_ (4)

t_C_ = 6 × (t_P_ + t_RX_) + t_BW_ (5)

n_C_ = 24/t_C_ (6)

V_BW_ = (t_BW_/3600) × 36 LMH × n_C_ (7)

R_BW_ = (V_BW_/Q_e_) × 100 (8)

where:

Q_e_: daily effluent production, throughput, L/d
J_net_: net flux, L.m^2^/h
J_ins_: instantaneous set flux, L.m^2^/h
A_m_: total membrane area, m^2^
V_BW_: daily backwash volume, L
R_BW_: backwash ratio, %
t_PT_: total production cycle duration in a day, h
t_CF_: time concentration factor
t_C_: one cycle duration, min
t_P_: production duration, 30 min
t_RX_: relaxation duration, 1 min
t_BW_: backwash duration, 15 s
n_C_: total cycles per day

**Figure 2:**
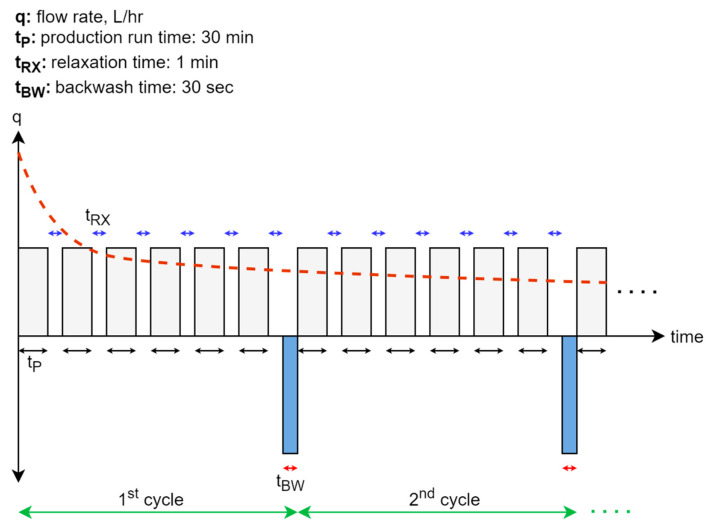
Conceptual cyclic membrane operation compared to continuous operation without relaxation and backwashing cycles (red line). In the continuous operation, a higher initial flux is achieved. However, rapid cake formation on the membrane causes the flux to decline.

The reactor was inoculated with biomass obtained from a local WW treatment plant’s anaerobic digester (Howard F. Curren Advanced Wastewater Treatment Plant; Tampa, FL, USA). The biomass had a volatile suspended solids (VSS) concentration of 10 g.L^−1^ and was screened through a 1.7 mm sieve to remove debris prior to inoculation. Biogas, reactor temperature, and permeate were continuously monitored using HOBO data loggers (Onset Computer Corporation; Cape Cod, MA, USA). To regulate the complex filtration cycles, a custom-made control system was designed using an Arduino microcontroller. Each membrane filtration cycle (t_C_ = 186.3 min) was comprised of a set of six 30-min permeate production runs (t_PT_ = 1386 min or 23.1 h) followed by a 60-s relaxation period (t_RX_), concluding with a 15-s permeate backwash (t_BW_) event (at 36 LMH flux) after each set of six runs (see Figure 2). Therefore, each cycle (t_C_) lasted for 186.5 min total with 7.7 cycles in a day (Equations (1)–(8)). The reactor hydraulic retention time (HRT) was set at approximately 0.8 day and operated at mesophilic conditions (35 °C ± 1). The flux was set to 4.5 LMH (J_ins_) throughout the experiment. The critical flux of the system was not experimentally determined. However, as the fouling rate was linear during the trial, it was concluded that our operation was below critical flux limits as described by Field et al. [18]. The CFV was set at 0.1 m.s^−1^ (0.5 L.min^−1^) while the backwash ratio (R_BW_) was 1.12%. The membrane feed was taken from the upper part of the reactor. The concentrate stream from the membrane module was returned to the bottom of the reactor where the synthetic sewage was fed. The membrane was chemically cleaned only once after the TMP exceeded 0.5 bar on the 183rd day of operation. The membrane was cleaned using a 500 mg·L^−1^ solution of NaOCl and 500 mg·L^−1^ citric acid by passing the solution through the module for 30 min each. Once chemical cleaning was performed, the membrane was rinsed with tap water prior to returning it to service. Except through sampling, no biomass was wasted.

### 2.2. Synthetic Sewage

Complex Organic Particulate Artificial Sewage (COPAS), derived from granulated dried cat food, was used to mimic real sewage as previously reported [19]. COPAS is composed of 92% volatile solids and 8% ash. The protein, carbohydrate, and lipid composition of COPAS are 40%, 43%, and 17%, respectively. These values compare well to the 40–50% carbohydrate, 40–60% protein, and 10% fat composition measured in domestic WWs [20]. The elemental composition of COPAS for carbon, nitrogen, and phosphorous were 52.5%, 6.35%, and 1.57%, respectively [19]. This is very close to the 50% carbon composition observed in domestic WW by Sotemann et al. [21] and provides a similar carbon to nitrogen ratio described by Ritmann and McCarty [22]. The COD and total solids (TS) of COPAS used were chosen as 501 mg·L^−1^ and 430 mg·L^−1^ to mimic medium-strength municipal WW characteristics (COD*_t_*/wt ratio, γ = 1.17) [23]. The influent had TN concentrations of 28.6 ± 2.9 mg·L^−1^ which were close to the average value of 35 mg·L^−1^ described in literature [23]. Influent TP concentrations were 13.6 ± 3.6 mg·L^−1^, which were within the 10–23 mg·L^−1^ range for domestic WW described by Henze et al. [24].

### 2.3. COD Mass Balance

The mass balance for COD was calculated using the daily influent, effluent, and biogas production. The daily influent loading of 501 mgCOD.L^−1^ corresponded to a 3.89 ± 0.37 g.day^−1^ loading rate (OLR was 0.61 ± 0.06 kgCOD.L^−1^.day^−1^). The daily average effluent COD concentration was 63.9 mg·L^−1^, which corresponded to 0.51 gCOD.day^−1^. The difference between the influent and effluent COD mass was 3.38 gCOD.day^−1^ which was assumed to be due to accumulation/growth and methane production. Since the average daily methane production was 0.525 L.day^−1^ at STP, the mass of COD routed towards biogas production was calculated using the standard value of 0.350 L.CH_4_.gCOD_removed_. Based on this theoretical methane potential, 1.5 g COD was used for methane production, while the remaining 1.88 g COD was either used for cell growth or accumulated in the reactor.

### 2.4. Monitoring Parameters and Analytical Methods

The reactor was operated for 7 months. During this period, the reactor soluble chemical oxygen demand (sCOD), total chemical oxygen demand (tCOD), solids, permeate tCOD, total nitrogen (TN), ammonia (NH_4_^+^-N), phosphorous (PO_4_^3−^—P), total organic carbon (TOC) concentrations, turbidity, TMP, biogas production, permeate production, and temperature were monitored. Solids were performed weekly according to Standards Methods [25]. All CODs, TN, NH_4_^+^-N, and TP were measured weekly using Hach HR digestion vials and Hach Test ‘N Tube^TM^ vials (Hach Company; Loveland; CO, USA). The reactor contents were centrifuged at 5000 RPM for 15 min and the supernatant was used to measure the sCOD. Bioags production was measured by a custom made wet tip mete and was corrected for standard temperature and pressure conditions (i.e., 0 °C and 1 atm). TOC and TN were measured using a Total Organic Carbon analyzer (Shimadzu TOC-V) coupled with a Total Nitrogen detector (Shimadzu TNM-1).

## 3. Results and Discussion

### 3.1. Membrane Performance

**Figure 3:**
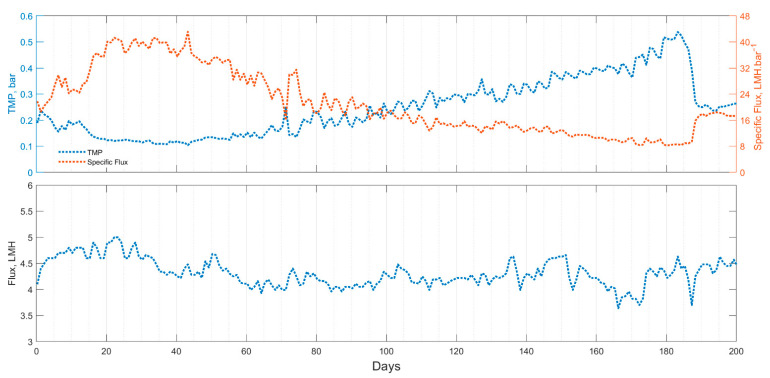
Membrane performance data of the upflow AnMBR: trans membrane pressure (TMP), specific flux; flux.

For the entire operational period, the membrane net flux was maintained at approximately 4.3 LMH corresponding to an HRT of 0.8 days. The TMP was stable at 0.15 bar for the first 60 days of operation (Figure 3). After 60 days, the TMP gradually increased and reached 0.5 bar on the 183rd day of operation. As a comparison, a similarly configured AnMBR, which was operated for 209 days but relied on gas-lift and periodic backwashing for fouling control, had to be cleaned three times after the TMP exceeded 0.2 bar (on days 19, 42, and 89) [26]. In addition, despite chemically cleaning the membrane multiple times, the TMP of that study stabilized at approximately 0.2 bar. The TMP of our experiment was around 0.25 bar, only slightly higher than the gas-lift study, and was achieved without chemical cleaning (Table 1). In another study, a gas-lift AnMBR was operated for 100 days with a CFV of 0.5 m.s^−1^ and with headspace gas used to continuously scour the membrane (ε, gas–liquid ratio value of 0.1) [10]. In that study, extensive weekly cleaning was performed consisting of relaxation, forward flushing with tap water, backwashing with tap water, forward flushing with NaOCl, and a final rinsing with tap water. Even though the membrane was maintained weekly by the above procedure, a decline in membrane flux was observed throughout the experiment. The initial flux of 18 LMH declined to 12 LMH after 24 h and an overall average flux of 10 LMH was maintained throughout the rest of the operation. These two studies show that gas-lift alone is not enough to mitigate membrane fouling and avoid frequent membrane cleaning. The amount of maintenance involved makes gas-lift less desirable for use in NSSS. The frequent chemical cleaning could also be problematic in terms of membrane integrity. Rabuni et al. showed that membrane cleaning with NaOH and NaOCl in PVDF membranes impacted the membrane stability causing a reduction in mechanical properties and chemical composition which was believed to negatively affect its hydrophilicity. This study also showed that the efficiency of membrane protein retention was compromised after physical and chemical cleanings [27]. Our study shows that cyclical relaxation and backwashing is a promising low-complexity and low-cost fouling mitigation strategy for external membrane modules.

Despite the short durations of relaxation and backwashing (3.12% and 0.13% of the daily overall cycle time, respectively), a sustainable membrane operation was achieved with a fouling rate of 0.17 kPa.day^−1^ (0.007 kPa.h^−1^ or 0.07 mbar.h^−1^). The change in fouling followed a linear trend throughout the entire trial indicating a subcritical flux operation. This fouling rate was significantly lower than the 1.0 kPa.day^−1^ recommended by Zsirai et al. and was similar to what Le-Clech et al. and Pollice et al. reported [28,29,30]. While the exact comparison is challenging (as published work on AnMBRs with external membranes is scarce), many submerged membrane systems have been studied with different configurations, reactor sizes, and membrane types. Pollice et al. [30] reported different fouling rates for both external and submerged systems varying from 0.02 to 12 kPa.h^−1^. From this study, the systems with the lowest fouling rates where all aerobic micro filtration systems (0.1 to 0.4 µm pore sizes). The only AnMBR in the study which had a 0.22 µm flat plate external membrane module had the highest fouling rate observed of 12 kPa.h^−1^. Martin-Garcia et al. [31] compared a flocculated AnMBR with a submerged membrane (F-AnMBR), and a granulated AnMBR with an external chamber and submerged membrane (G-AnMBR) in a pilot-scale study. They operated these two reactors in parallel and tested their contents in short-term fouling experiments using a gas-lift external UF membrane module. They reported a critical flux of 4 LMH for both scenarios. The fouling rate was much higher for the F-AnMBR (24–150 kPa.h^−1^) system than the G-AnMBR (6–12 kPa.h^−1^) when the flux was set at 11–12 LMH. They also reported that the fouling declined from 70.8 kPa.h^−1^ to 1.56 kPa.h^−1^ in the F-AnMBR system when the superficial gas velocity was increased from 0.02 to 0.21 m.s^−1^. These studies indicate that our fouling rate was an order of magnitude lower than the lowest fouling rate presented by AnMBRs in literature and within the range of AeMBRs.

Studies that compare different fouling behaviors often use short-term experiments from several hours to a couple of days. While this method can give an idea on how the system will perform in long-term operations, it is generally considered controversial especially for the determination of critical flux and fouling prediction. The fouling buildup in membrane systems behave differently in long-term operations at lower fluxes than experiments with short-term operations at higher fluxes [32]. This might explain the large difference between fouling rates in the previous studies and our study. More recently, another study investigated the short-term effects of fouling on an external gas-lift and a submerged membrane module [33]. At 15 LMH, the submerged system surpassed the gas-lift in 7 days of operation achieving 0.009 kPa.h^−1^ fouling rate compared to 0.047 kPa.h^−1^. The submerged system in this study achieved a similar fouling rate to our operation. However, as mentioned earlier, short-term experiments might not necessarily follow parallel trends in long-term operations.

Our fouling rates were mostly lower when compared to recent biological techniques used for fouling mitigation such as quorum quenching and the population control of filamentous microorganisms. Quorum quenching aims to disrupt the accumulation of signaling molecules used by mutually growing microorganisms, thus decreasing the concentration of extracellular polymeric substances (EPS) [34]. EPS are considered as one of the major contributors for the membrane fouling; therefore, the reduction of EPS can improve membrane performance [35]. Oh et al. tested quorum quenching with a recombinant *E. Coli* in an AeMBR using a submerged PVDF hollow fiber membrane, and achieved substantially lower fouling rates compared to the control reactor [36]. After operating the reactor at 18 LMH for 8 days, this test observed a fouling rate of 0.047 kPa.h^−1^. In another study, a commercial enzyme, porcine kidney acylase 1, was used for quorum quenching in a lab-scale submerged AeMBR [37]. In addition to air scrubbing, the membrane was relaxed for 3 min after 9 min of permeation. During the 113 h of operation, a fouling rate of 0.075 kPa.h^−1^ was achieved, which was approximately 8 times lower than the control reactor. Compared to our results, this fouling rate was approximately 3 times higher even after considering the flux differences between the two studies. Similar fouling results were found in another study using a ceramic membrane and a consortium of facultative quorum quenching microorganisms for the Acyl-homoserine lactones (AHLs) degradation in an AnMBR [38]. After 14 days of operation at 7.2 LMH, the reactor with quorum quenching microorganisms had a fouling rate of 0.074 kPa.h^−1^ while reaching a TMP of 30 kPa. The control reactor reached the same TMP in 7 days. It should be also noted that the fouling was almost zero until the 12th day of operation. Quorum quenching could be coupled with other physical/chemical methods to further improve membrane performance. However, the isolation, cultivation, and encapsulation of the bacteria or the enzymes used for quorum quenching increased the initial and operational complexity of the system. From this perspective, our operation reduced the overall system complexity by relying on existing hardware to reduce membrane fouling.

Similar to quorum sensing, filamentous microorganisms can increase the amount of soluble microbial products (SMP) and cause irreversible fouling [39]. It was also found that increased growth of filamentous bacteria forms a non-porous cake layer on the membrane surface, further increasing the membrane fouling [40]. However, positive effects of filamentous bacteria are also found in the literature when the population is not in excessively high concentrations [41,42]. Banti et al. studied the effects of different dissolved oxygen concentrations and food to microorganisms (F/M) ratios on the filamentous bacterial population and their effects on membrane fouling rates [43]. The reactor TMP was stable at 2.3 ± 0.2 kPa during the initial 90 days when the F/M ratio was lower than 0.5 gCOD/gMLSS.d^−1^. Excessive filamentous bacteria growth was inhibited, which resulted in the reduction of SMP and irreversible fouling. Compared to our operation, this study showed a lower fouling behavior under lower loading rates. While the TMP of our reactor was also stable around 15 ± 0.4 kPa during the initial 90 days, their system was operated at approximately 3 times higher flux rates than our system. It should also be noted that our average F/M ratio was around 0.39 gCOD/gMLSS.d^−1^ which falls in less than the 0.5 gCOD/gMLSS.d^−1^ observed in their operation. From an operational perspective, filamentous population control could help AnMBR fouling mitigation, especially considering that they were the dominant form of bacteria in the sub-visible particle range (0.45–10 µm) and the main cause of fouling in an AnMBR operated at similar loading rates (F/M ratio 0.47 gCOD/gMLSS.d^−1^) [44]. However, when the F/M ratio was higher than 0.5 gCOD/gMLSS.d^−1^, the TMP rapidly increased resulting in a 0.042 kPa.h^−1^ fouling rate [43]. Therefore, for high strength WWs, control of the filamentous microbe population might be challenging to enact, especially with NSS systems.

A similar result to our system was reported by Verhuelsdonl et al. [45] in a 329-day operation with a pilot-scale submerged AeMBR. The system used relaxation, backwashing, and air bubbling to prevent fouling. They reported a fouling rate of 0.003 kPa.h^−1^ for the entire operation period, which is 50% lower than what we observed in our study. This lower fouling rate could be explained by several factors. First, AeMBRs tend to have lower fouling rates compared to AnMBRs. In a study comparing an AeMBR and an AnMBR, both with submerged microfiltration membranes, the anaerobic system had a fouling rate almost twice that of the aerobic reactor (0.063 and 0.037 kPa.day^−1^, respectively) [46]. This is predominantly due to the greater presence of micro-particles exhibiting a much higher specific filtration resistance in the AnMBR than in the aerobic system. Another reason is the membrane type used for the filtration. Polyethersulfone (PES) has higher permeability and thus shows lower fouling propensities compared to PVDF membranes. Several studies tested different membrane materials and reported that PES is superior in terms of fouling formation [47,48]. Finally, relaxation and backwashing duration also affect fouling rates. In our study, only 3.33% of the overall daily cycle time was used for the RX and BW while they reported a 20% daily off time for their RX time (1-min RX followed by 4-min filtration). While a longer duration of relaxation had a negative effect on permeate productivity, the membrane foulants had a chance to dislodge from the membrane surface. Considering the membrane material, the anaerobic reactor contents, and the externally positioned membrane, it is apparent that the fouling mitigation techniques used in this paper resulted in a very low fouling rate and significantly increased the duration between chemical cleanings of the membrane.

**Table 1:** Literature comparison between the experimental conditions and fouling results of this experiment and cited literature.

References	System Type, Size, and HRT	Influent Characteristics, mg/L	Membrane Characteristics	Initial Flux, LMH	Fouling Rate, kPa/h	Flux Reduction	Fouling Mitigation Methods
This study	Anaerobic; 6.2 L; 0.8 days	Synthetic; TSS: 430; COD: 501; TN:28.6; TP:13.6	0.03 µm; 0.075 m^2^; external tubular PVDF	4.5	0.007	-	CFV: 0.1 m/s; frequent BW; frequent RX
Prieto et al., 2013 [10]	Anaerobic; 10 L; 3 days	Synthetic; TSS: 520; TOC: 504; COD: 1260; TN: 54; TP:44	0.03 µm; 0.013 m^2^; external tubular PVDF	18	-	18 LMH to 10–15 LMH in 100 days	gas-lift; CFV: 0.5 m/s; weekly PH and CH cleaning
Dolejs et al., 2017 [26]	Anaerobic; 10 L; 30–36 h	Synthetic; TSS: 400; COD: 1000	0.03 µm; 0.066 m^2^; external tubular PVDF	4.5	-	4.5 LMH, no reduction	gas-lift; CFV: 0.1 m/s; frequent BW; CH cleaning on day 19, 42, 89
Pollice et al., 2005 [30]	Aerobic; 20 L; 3.3 h	Domestic and synthetic; COD: 850	0.03 µm; 0.5 m^2^; submerged hollow fibers	12	0.02	-	air scrubbing; frequent BW and RX; frequent CC
Pollice et al., 2005 [30]	UASB; NS *; NS	NS; COD: 10213	0.22 µm; external chamber submerged flat-sheet PVDF	30	12	-	air scrubbing; cleaning but NS
Martin-Garcia et al., 2011 [31]	Flocculated Anaerobic; 38 L; 16 h	Domestic; SS: 84; COD: 338; BOD5: 167; ammonia: 35	0.03 µm; external tubular PVDF	11–12	24–150	-	gas-lift; SGD: 0.2–1.2 m^3^/h
Martin-Garcia et al., 2011 [31]	Granulated Anaerobic; 38 L; 16 h	Domestic; SS: 84; COD: 338; BOD5: 167; ammonia: 35	0.03 µm; external tubular PVDF	11–12	6–12	-	gas-lift; SGD: 0.2–1.2 m^3^/h
Martinez et al., 2020 [33]	Anaerobic; 20 L; NS	NS	0.04 µm; 0.93 m^2^; submerged hollowfiber PVDF	15, 20, 25	0.009	-	gas-lift; SGD: 1–1.2 m^3^/h
Martinez et al., 2020 [33]	Anaerobic; 20 L; NS	NS	0.04 µm; 0.93 m^2^; external tubular PVDF	15	0.047	-	CFV: 0.51 m/s; SGD: 0.3–0.4 m^3^/h
Oh et al., 2012 [36]	Aerobic; 1.2 L; 12 h	Synthetic; glucose, 400; yeast extract, 14; bactopeptone, 115; (NH_4_)_2_SO_4_, 104.8; KH_2_PO_4_,	NS; 86 cm^2^; submerged hollowfiber	18	0.047	-	air scrubbing; quorum quenching with recombinant E. coli
Jiang et al., 2013 [37]	Aerobic; 3 L; 4.8 h	500 glucose; 2500 yeast extract; 25 bactopeptone; 250 (NH_4_)2SO_4_; 150 K_2_H_2_PO_4_; 150 KH_2_PO_4_	0.01 µm; 0.07 m^2^; submerged hollow fibers	12	0.075	-	air scrubbing; frequent RX; quorum quenching with porcine kidney acylase 1 enzyme
Xu et al., 2020 [38]	Anaerobic; 4.5 L; NS	Domestic	NS; NS; external chamber submerged	7.2	0.074	-	quorum quenching with Acyl-homoserine lactone enzymes
Verhuelsdonk et al., 2021 [45]	Aerobic; NS; NS	Brewery WW; COD: 512; BOD5: 124; NH_4_-N: 45; PO_4_-P: 9.6	0.038 µm; 108 m^2^; submerged rotating PES	9.5–11.5	0.003	-	air scrubbing; frequent RX; frequent BW

* NS: not specified.

### 3.2. Treatment Performance

The AnMBR system showed a stable performance in terms of COD removal, achieving 85 ± 8.9% for the entire operating period. The COD removal efficiency was comparable, although on the lower end of the removal efficiencies presented in a recent review of pilot-scale AnMBRs treating domestic WW. The pilot-scale systems described removal efficiencies ranging from 85–93.7% [49]. Lab-scale systems treating synthetic WW tend to have COD removal efficiencies over 95%, as the synthetic feeds are readily biodegradable and process control is easier to implement at the bench scale [50]. The use of COPAS for the system feed increased the complexity of the feed compared to traditional synthetic feeds and better represented domestic WW, as indicated by the COD removal efficiency matching the values presented by the pilot systems treating domestic WW. The average permeate COD concentration was around 63.9 ± 31.1 mg·L^−1^. The permeate quality is comparable to the previous GI-AnMBR studies which also treated COPAS [10,26]. These studies reported that their average CODt concentrations were 55 ± 18 mg·L^−1^ and 75 ± 34 mg·L^−1^ in the final permeate. The permeate COD concentrations were similar to a pilot-scale system treating real domestic WW operated under similar conditions including an average influent COD concentration of 445 mg·L^−1^ and operated with an HRT of 0.9–1.2 days [51]. This pilot-scale system had an average permeate COD concentration of 77 mg·L^−1^. This is further evidence of both the adequate treatment performance of the AnMBR as well as the suitability of COPAS as a WW surrogate.

**Figure 4:**
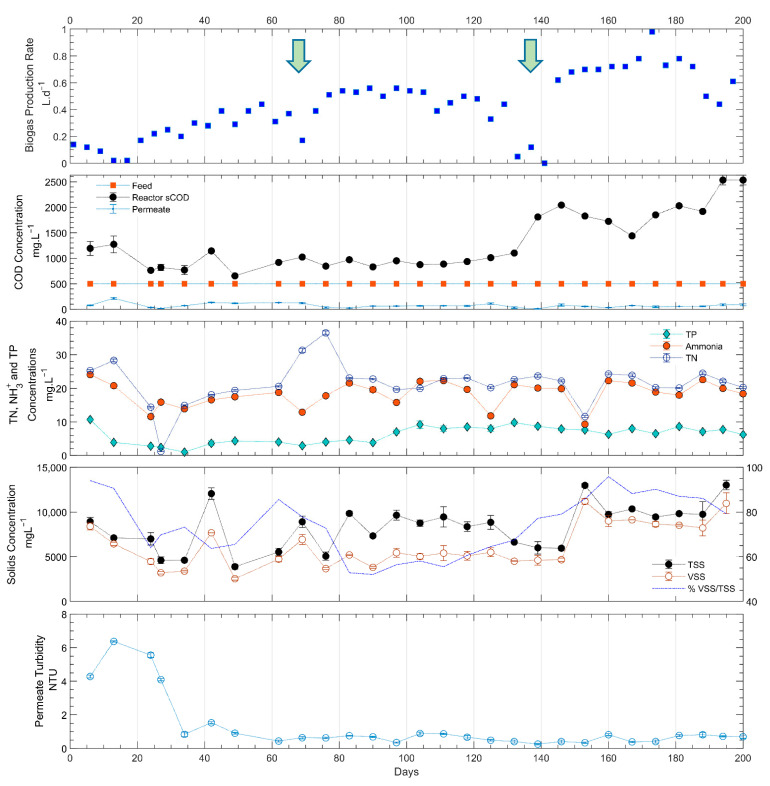
Operating and performance data for the upflow AnMBR: biogas production rate (arrows indicate when sensor cleaning was conducted); tCOD concentration of influent, permeate, and sCOD concentration of reactor content; TN, TP, and ammonia concentration.

Membrane filtration was the primary reason for consistent permeate COD concentrations as UF retains all suspended and colloidal solids and only soluble fractions pass through the membrane. Filtration was able to deliver consistent permeate quality, despite the steady increase of sCOD concentrations within the reactor which was observed throughout the testing period (Figure 4). The turbidity in the permeate was consistently lower than 1 NTU (representing 99.9% removal) after the start-up period (0–50 days) while a higher turbidity of 6.9 ± 2.3 NTU was reported in the Gl-AnMBR study [10]. Overall, AnMBR in this study had high COD removal rates and was able to consistently produce a low turbidity effluent. 

### 3.3. Nutrients and Solids

TN, ammonia NH_3_^+^, and TP concentrations were stable and 22.7 ± 5.1, 18.8 ± 3.7, and 6.9 ± 2.0 mg·L^−1^, respectively (Table 2). Even though ammonia was not present in the influent, the mineralization of proteins in COPAS resulted in ammonification, leading to the presence of ammonia in the permeate. Anaerobic digestion on its own does not remove ammonia; the removal of ammonia requires subsequent steps of either nitrification/denitrification or anammox [23]. Struvite (magnesium ammonium phosphate) precipitation is a purely chemical process that can remove nutrients in WW treatment. While it can be used to reduce ammonia concentrations in the permeate, the formation of struvite could also develop in the membrane pores. However, this could be problematic for membrane performance. In AnMBRs, it was reported that struvite formation could be responsible for internal membrane fouling and cake hardening [4,52]. The TN removal efficiency was 20.6%. However, the majority of TN in the permeate was ammonia (>95%). This means that only a small portion of nitrogen was used either for cell growth or participated in struvite precipitation and the remaining was converted to ammonia. The ammonia could be used for resource recovery strategies such as fertigation since the permeate is free of solids and pathogens. TP removal was slightly higher than the TN, achieving a 49.3% removal. As with ammonia, phosphorous is a component of struvite and could be precipitated within the reactor or on the membrane surface. Phosphorous can also precipitate as other minerals such as calcium phosphate.

The reactor total suspended solids (TSS) and VSS concentrations initially decreased from 10 g.L^−1^ of the initial seed concentration to 5 and 4.5 g.L^−1^, respectively. However, after the start-up period, TSS concentration increased to around 10 g.L^−1^ while VSS concentration stayed around 5 g/L until the 160th day of operation. The initial decrease was presumably due to the settling of the solids in the reactor. Even though the membrane concentrate was recirculated back into the inlet of the reactor at 0.5 L.min^−1^, a sludge blanket formed near the bottom of the reactor resulting in lower TSS at the top of the reactor (where the reactor sample was collected). As samples were taken at the top of the reactor, the solids and sCOD concentrations were affected by the sludge blanket movement within the reactor. It was observed that any disruption to the sludge blanket would result in temporary fluctuations of solids and COD concentrations in the reactor sample (Figure 4). The TSS concentration in the reactor exceeded 10 g.L^−1^ a few times during the operation. This indicates that the recalcitrant portion of the COPAS (43%) as well as the accumulated biomass within the reactor appeared in the reactor samples when a disruption was caused in the reactor’s settling zone.

### 3.4. Biogas

The average biogas production rate after the first 50 days was 0.75 ± 0.44 L.day^−1^ (0.122 L.gVSS^−1^ or 0.156 L CH4.gCOD_removed_^−1^). The methane content of biogas from AnMBR is generally between 55–75% [23,53]. For this experiment it was assumed that the biogas contained 70% methane. Compared to the theoretical methane yield of 0.350 L CH4.gCOD_removed_^−1^, the production was lower than the theoretical, but closer to values reported in literature for pilot-scale systems. Robles et al. reported 0.193 L CH_4_.gCOD_removed_^−1^ in their pilot-scale AnMBR treating domestic WW; however, their study concluded that the low methane production rate was due to sulfate reduction [54]. Galib et al. also reported a similar value of 0.18 L CH_4_.gCOD_removed_^−1^ when loading a reactor with an OLR of 0.4 kgCOD.L^−1^.day^−1^ [55]. It should also be noted that the COPAS used in this research was only 57% biodegradable [19]. Therefore, the theoretical maximum methane potential was 0.200 L CH_4_.gCOD_removed_^−1^, which is much closer to our 0.156 L CH_4_.gCOD_removed_^−1^ result. The remaining 22% volume could be either in the form of dissolved methane in the permeate or variations in methane content in the biogas. The lower biogas production on the 70th and 130th days of operation was due to reactor headspace leaks resulting from sensor maintenance.

The increase in biogas production that occurred after the maintenance event on the 130th day was likely due to a disturbance of the reactor biomass. This disturbance can also be seen by the rapid increase in the sCOD and TS concentrations which occurred after day 130 (Figure 4). The sensor maintenance was accomplished by stopping all the pumps and the heating element. This event somehow disturbed the settled biomass and caused higher suspension at the top of the reactor. In addition, the heating element temperature sensor was placed in the middle of the reactor. Therefore, by the time the entire reactor temperature reached 35 °C, the biomass closer to the heating element was most likely heated beyond the desired temperature setpoint. This could have triggered biomass disintegration after a level sensor maintenance since it had been observed in the reactor each time after a level sensor maintenance was conducted. Compared to the Gl-AnMBR study, biogas production rate was slightly lower. Prieto et al. reported an average of 450 L.m^−3^ sludge per day of biogas production (0.280 L CH_4_.gCOD_removed_^−1^) [10]. One reason for the lower biogas production in this study could be that our reactor was operated at an HRT of 0.8 day and 501 mgCOD.L^−1^ influent whereas Gl-AnMBR was operated at an HRT of 3 days and 1250 mgCOD.L^−1^ influent. The higher HRTs could potentially increase the hydrolysis rate which enables higher methanogenic activity. 

**Figure 5:**
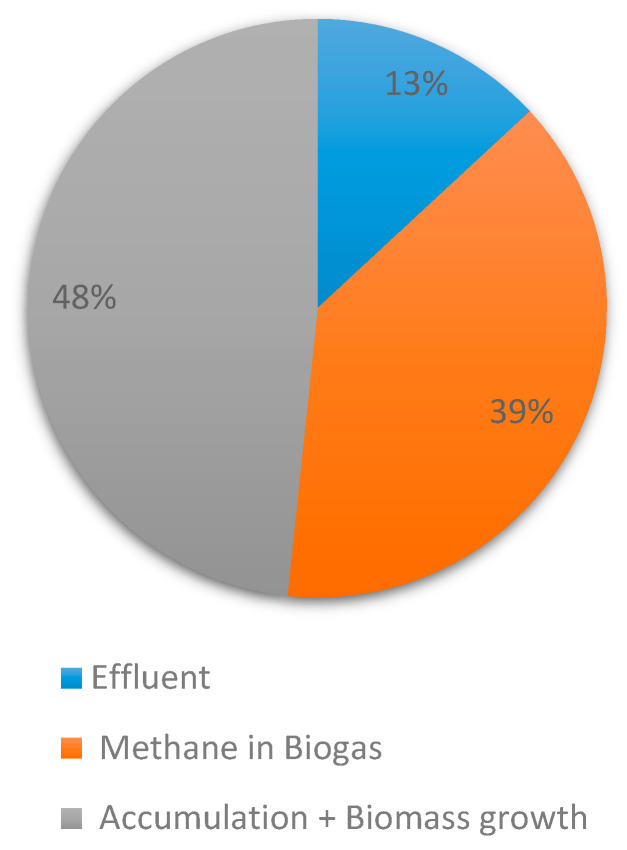
COD mass balance for the entire operation (assuming 70% methane in biogas).

The COD mass balance showed that 48% of the COD was retained in the reactor while 39% converted to methane and 13% was left in the permeate (Figure 5). The COD mass balance was comparable to those reported with similar OLRs in different studies. Galib et al. reported in their COD mass balance that 33.3% of the COD was routed towards methane, 10.5% towards dissolved methane, 20.3% left in the permeate or waste, 12% accumulated as biomass, and 24% accumulated in the reactor. Typically with AnMBRs operated at mesophilic conditions, methane dissolved in the permeate ranges of 11–33% [56]. Therefore, by assuming 10–15% of dissolved methane was found in our permeate, our COD mass balance became quite similar to Galib et al.’s results.

### 3.5. NSSS Comparison

A comparison of the system treatment performance and ISO 30500 standard is presented in Table 3. Due to the use of synthetic wastewater, biological pathogen parameters were not evaluated. The average effluent quality of this system was close to meeting the WW treatment criteria established by the ISO standard. ISO 3500 sets the COD threshold for discharge into surface waters or other restricted urban uses (Category B waters) as 150 mg·L^−1^, which is well above the average AnMBR effluent COD concentrations. It should be noted that the UF membrane alone was able to reduce COD concentrations as high as 2500 mg·L^−1^ in the AnMBR to below 100 mg·L^−1^. The additional removal of organics by the membrane resulted in a high-quality permeate which helped meet the ISO 30500 Category B criteria for both the COD and TSS concentrations. The permeate had little to no TSS and was undetected in our analysis throughout the operational period. Thus, for TSS, the system was able to meet the more stringent Category A water which can be used for unrestricted urban uses. Although not part of the ISO 30500 criteria, the effluent turbidity was consistently lower than 1 NTU which would be impossible to achieve without membrane filtration. The AnMBR did not achieve the nutrient reduction limits set forth by ISO 30500. The guidelines require a 70% and 80% removal of TN and TP, respectively, which is significantly higher than the 20.6% and 49.3% reductions of TN and TP achieved by the system. However, this was expected as anaerobic digestion alone has very few mechanisms in place for nutrient removal. To satisfactorily meet the ISO 30500 criteria, the AnMBR would have to be coupled with additional downstream processes for nutrient removal or recovery.

## 4. Conclusions

Our study shows that cyclical operation with frequent relaxation (3.2% of the daily overall cycle time) and periodic backwash (0.13% of the daily overall cycle time) is a sustainable approach to enable prolonged membrane operation, albeit at lower flux, without requiring an invasive cleaning (183 days at 4.5 LMH). The cyclic operation resulted in a very low fouling rate of 0.007 kPa.h^−1^ and significantly increased the duration between chemical cleanings of the membrane. Even with a lack of comparable data to external systems, our operation has achieved one of the lowest fouling rates in the literature. This operation could potentially benefit NSSS due to its low complexity, maintenance, and energy requirements. Compared to Gl-AnMBRs, which use headspace gas for membrane scrubbing, this system maintained a lower TMP for a longer period. This study also shows that AnMBR technology can treat medium-strength WW, achieving a desirable removal efficiency in terms of biological degradation and throughput (>86% COD removal at 0.8-day HRT) and higher quality effluent (<1 NTU), which is suitable for resource and energy recovery.

## Figures and Tables

**Table 2:** Summary of average performance data of upflow anaerobic membrane bioreactor.

	AVG	*n*
Temperature, C	34.6 ± 1	-
Trans membrane pressure (TMP), bar	0.25 ± 0.11	4872
Daily permeate production, mL	7647 ± 1238	200
Biogas production, L/day	0.75 ± 0.44	150
Net flux (J_net_), LMH	4.3 ± 0.7	-
Specific flux, LMH/bar	20.9 ± 10.7	-
pH	6.72 ± 0.3	75
Total solids, mg/L	9742 ± 3876	27
Volatile solids, mg/L	6253 ± 2707	27
Total suspended solids, mg/L	8284 ± 2479	27
Total volatile suspended solids, mg/L	6165 ± 2385	27
Total chemical oxygen demand removal efficiency, %	85.8 ± 8.9	27
Permeate Quality (first 50 days excluded)		
Total chemical oxygen demand, mg/L	63.9 ± 31.1	20
Total organic carbon, mg/L	11.3 ± 10.6	20
Ammonia, mg/L	18.8 ± 3.7	20
Total phosphorous, mg/L	6.9 ± 2.0	20
Total nitrogen, mg/L	22.7 ± 5.1	20
Turbidity, NTU	0.6 ± 0.2	20

**Table 3:** Summary of the system performance compared to ISO 30500 liquid effluent standards for chemical parameters over the pseudo-steady state period (first 50 days excluded) *.

Parameters	Influent	Effluent	% Reduction	ISO 30500 (CAT A/B) **
TSS	198.4 ± 12.5	ND ***	100	10/30
tCOD (mg/L)	501 ± 43	63.9 ± 31.1	87.2 ± 6.2	50/150
pH	6.65 ± 0.2	6.72 ± 0.3	-	6–9
TN (mg/L)	28.6 ± 2.9	22.7 ± 5.1	22.6 ± 13.7	70% reduction
TP (mg/L)	13.6 ± 3.6	6.9 ± 2.0	49.3 ± 14.7	80% reduction

*: pathogen parameters were not evaluated as part of this study. **: CAT A effluent can be used for unrestricted urban uses, CAT B effluent can be discharged or used in restricted urban uses. ***: not detected.
